# Predicting Well-Being Among the Elderly: The Role of Coping Strategies

**DOI:** 10.3389/fpsyg.2020.00616

**Published:** 2020-04-03

**Authors:** Laura Galiana, José M. Tomás, Irene Fernández, Amparo Oliver

**Affiliations:** Department of Methodology of the Behavioural Sciences, University of Valencia, Valencia, Spain

**Keywords:** problem-focused coping strategies, emotion-focused coping strategies, psychological well-being, subjective well-being, elderly

## Abstract

**Objectives:**

This study aims to offer a wider view on the role of coping strategies on elderly’s well-being by means of literature-based competitive structural equation models (SEMs).

**Methods:**

857 older adults were surveyed. Measures included Ryff’s scales of Psychological Well-being and the Coping Strategies Questionnaire. Competitive SEMs were tested.

**Results:**

In the retained model, the religious coping dimension was removed, and the remaining dimensions were defined by problem- and emotion-focused coping, which explained both psychological and subjective well-being factors (χ^2^(46) = 165.910, *p* < 0.001; CFI = 0.906; GFI = 0.957; RMSEA = 0.058 [0.048,0.067]).

**Discussion:**

Results pointed to the relevance of coping strategies for achieving adequate well-being, with emotion-focused coping strategies being the ones with negative and highest predictive power over the two dimensions of well-being. Interventions aiming at improving old people’s well-being should, put their focus on decreasing the use of emotion-coping strategies.

## Introduction

Well-being may be defined as ‘the striving for perfection that represents the realization of one’s true potential’ (Ryff, 1989, p. 100). However, well-being has been conceptualized in several forms. Ryan and Deci (2001) distinguished between two perspectives from which well-being can be understood: hedonic and eudaimonic. The former states that well-being consists of pleasure attainment and pain avoidance, and could be equated to happiness (Bradburn, 1969). The latter focuses on self-realization and meaning and conceptualizes well-being as the degree of functioning a person is capable of achieving (Ryff, 1989; Ryan and Deci, 2001; Waterman et al., 2010). Hedonic well-being has been traditionally associated to subjective well-being (SWB) (Keyes et al., 2002), while eudaimonic well-being has been identified as psychological well-being (Extremera et al., 2011). Subjective well-being, then, better accounts for the evaluation of one’s life in terms of satisfaction and happiness. Psychological well-being, in turn, is best when referring to self-fulfillment and personal functioning.

Ryff (1989) compiled the theoretical conceptualizations of psychological well-being into six dimensions: self-acceptance, positive relations with others, autonomy, environmental mastery, purpose in life, and personal growth. These dimensions explain short-term well-being as well as more enduring life challenges that also constitute the sense of well-being. These dimensions can be measured with the Psychological Well-being Scales (Ryff, 1989; Ryff and Keyes, 1995), which represent one of the most widely used models of well-being (Tomás et al., 2012). However, there is a lack of consistency in the understanding of how these six dimensions articulate into a construct. While Ryff (1995) and Ryff and Keyes (1995) thought the six dimensions were indicators of a single construct of psychological well-being, Keyes et al. (2002) have more recently argued that there are strong associations among some of the six dimensions in Ryff’s model with subjective well-being. Specifically, evidence shows a strong association between self-acceptance and environmental mastery (Keyes et al., 2002).

A great amount of literature has focused in the understanding of variables which may explain these dimensions, and therefore promote humans’ optimal functioning. Ryan and Deci (2001) identified several potential antecedents of well-being, such as personality traits, emotions or social support. Some other authors have posed coping as one of the factors that can determine well-being (Cicognani, 2011; Por et al., 2011). Coping is defined as a set of cognitive and behavioral efforts that aim at managing the specific demands that exceed the resources of the individual (Lazarus and Folkman, 1984; Carver et al., 1989). The goal of coping strategies is to compensate or alleviate stressful situations by means of either the reformulation of objectives or the adjustment to a new and positively assessed situation (Gamrowska and Steuden, 2014). This compensation can be achieved by two means: emotion- and problem-focused coping strategies. Emotion-focused coping involves managing the emotional distress that is associated with a problematic situation (Lazarus and Folkman, 1984), this coping strategy involves individual’s self-regulation aiming at minimizing emotional consequences of stressful situations (Schoenmakers et al., 2015). Problem-focused coping is centered toward modifying the problem at hand, and comprises individual actions undertaken to deal with stressful situations in order to change a situation that presents stress to the individual (Lazarus and Folkman, 1984; Schoenmakers et al., 2015). According to Folkman (1984), problem-focused coping strategies are more commonly implemented in situations in which the problem can be altered, and they help maintaining psychological well-being. Emotion-focused coping strategies, instead, are to be used when the problem is inalterable. Although, the distinction of emotion and problem-focused coping strategies is widely employed (Baker and Berenbaum, 2007; Dardas and Ahmad, 2015), it has also been argued that relying completely on these two general categories of coping strategies may hide important differences within categories, and also distorts the fact that these categories are not independent, with different coping strategies overlapping and facilitating each other (Folkman and Moskowitz, 2004). For example, seeking social support may be included in emotion- or problem-focused coping depending on its particular nature. For example, Schoenmakers et al. (2015) considered that improving relationships is a problem-focused way of coping and can be achieved by making new friends or re-establishing contact with old ones. Nevertheless, coping strategies can be related to well-being, by alleviating stressful situations. In this line, Tovel and Carmel (2014) have found a positive relation between coping strategies and measures of old people’s subjective well-being. Tomás et al. (2012) also posed a relation among problem-coping, emotion-coping, and resilience coping, altogether predicting old adults’ well-being. Phelan et al. (2004) identified coping as one of their four factors in their model of successful aging. Yet another study offered evidence of the role of problem- and emotion-focused coping strategies as predictors of well-being in a sample of young adults (Mayordomo-Rodríguez et al., 2015a). They found a positive direct effect of problem-solving coping and a negative effect of emotion-based coping on well-being. They also found that religion was not a relevant coping strategy, and that two coping strategies (problem-solving focus and avoidance) showed cross-loadings in the two factors modeled (problem- and emotion-based coping). This model considered Ryff’s six dimensions as indicators of a single well-being factor.

The need to establish the predictive power of coping strategies on well-being in old people is well endorsed by many theories of aging. For instance, the Selective Optimization with Compensation (SOC) model (Baltes and Baltes, 1990) or the Preventive and Corrective Proactivity (PCP) model (Kahana et al., 2014). The SOC model links well-being to coping strategies, as orchestrating processes that lead to outcomes such as loss minimization, growth, or well-being (Baltes and Baltes, 1990). The SOC model is essentially a model of problem-focused coping strategies, because selecting and optimizing goals and priorities, and trying to compensate for losses in energy and resources are all focused on direct actions (Wiese et al., 2000; Freund and Baltes, 2002). The PCP model states that older adults are very likely to face normative stressors such as chronic illnesses, social losses, and a lack of person–environment fit. The model also states that these stressors may be successfully handled with the use of internal and external resources. Among the internal resources, Kahana et al. (2014) stressed the importance of coping strategies, which are resources that can be translated into proactive behavioral adaptations (health promotion, helping others, planning ahead, marshaling support, or role substitution). When it comes to behavioral adaptations, it seems that problem-focused coping strategies would be more facilitators of behavioral change, as they imply problem-oriented actions, instead of self-regulation (Schoenmakers et al., 2015). These adaptations finally may exert an effect on quality of life and well-being. Therefore, the PCP model considers coping an internal resource directly linked to successful aging, as marked by well-being, and taking into account this approach, problem-focused strategies would be those to endorse for successful aging outcome. However, evidence points that older adults use more emotion-focused coping strategies, rather than problem-focused ones (Mayordomo-Rodríguez et al., 2015b; Chen et al., 2018).

All in all, literature points coping strategies both in general population and, specifically, in the older population. It has to be kept in mind that aging itself is a process of many changes, including not only physical, but also psychological and social changes (i.e., retirement, income decrease, or empty nest). As such, it is of paramount importance for older people to dispose of adequate strategies in order to minimize the emotional consequences of such stressful situations and adequate adapt to them (Schoenmakers et al., 2015). Because of that, the study of coping strategies can be the clue for the desired successful aging.

Although a considerable amount of research on adults’ well-being and coping strategies has been published, not much regards the relationship between them (Tomás et al., 2012). Taking into account aging of the population, scientific knowledge on determinants of old people’s well-being could be a good starting point for the development of future interventions in this population. In Spain, for example, older population has grown in later years and it is expected to further grow in future years due to the fact that between 1957 and 1977, the so-called baby-boom generation took place, and this generation has currently started reaching retirement age (Serrano et al., 2014). Increasing aging of the population is also a fact for Europe in general (Długosz, 2011; Liotta et al., 2018). Indeed, the improvement of older people well-being through psychological variables that can be taught and learned in the old age, such as coping strategies, would enable the development of low-cost intervention programs and strategies.

This study therefore aims to offer a wider view on the role of coping strategies on elderly’s well-being, by testing literature-based competitive structural equation models (SEMs). General hypotheses are: (a) problem-focused coping will have a positive effect on well-being; and (b) emotion-focused coping will have a negative effect on well-being; regardless the conceptualization of well-being. The emotion and problem-focused coping strategies are based on results by Mayordomo-Rodríguez et al. (2015a). These hypotheses are supported by meta-analytic evidence from Penley et al. (2002).

## Methods

### Procedure and Participants

The study consisted of a panel design in which older adults attending long life learning programs of the University of Valencia (Educational Programs for Older People, La Nau Gran), Spain, were surveyed. La Nau Gran is the University of Valencia Program for older people, and it depends on the Vice-Rectorate of Educational Policy and Training. The allows adults of advancing age people to participate as students in the University of Valencia, sharing the classrooms and subjects with the rest of the students who attend the regulated degrees. Approval of the Ethics Committee of the University of Valencia was obtained (reference 2014/H1403533342121), and participants were asked to give their informed consent. Those who were willing to participate were surveyed, with a response rate of 77.54%.

The sample consisted of 857 individuals, aged between 60 and 92 years old. 69.2% of the sample were women. Regarding marital status, 45.7% were married, 28% single, 20.4% widow/widower, and 5.8% divorced. Descriptive statistics of all the quantitative variables under study are in Table 1.

**TABLE 1:** Means, standard deviations, minimum and maximum of the quantitative variables under study.

Statistic	Age	AUT	EM	PG	PRO	PL	SA	NSF	PR	AVD	REL	PSF	OEE	SSS	Health
Mean	68.24	3.48	3.51	3.75	3.43	3.21	3.50	2.47	3.49	2.94	2.50	3.67	2.04	3.17	3.92
*SD*	5.93	0.56	0.57	0.60	0.66	0.70	0.65	0.75	0.69	0.87	1.24	0.68	0.75	0.88	0.75
Minimum	60	1	1	1	1	1	1.67	1	1	1	1	1	1	1	1.29
Maximum	92	5	5	5	5	5	5	5	5	5	5	5	5	5	5

*EM, environmental mastery; PG, personal growth; PRO, positive relations with others; PL, purpose in life; SA, self-acceptance; NSF, negative self-focus; PR, positive reappraisal; AVD, avoidance; REL, religion; PSF, problem-solving focus; OEE, overt emotional expression; SSS, social support seeking; AUT, autonomy.*

The procedure involved self-administered surveys. Participants answered the surveys in their classrooms. The interviewers were trained for these particular surveys. The session to fill out the surveys took an average of 30 min.

### Measures

Three scales among the information gathered on the surveys were used:

(a) Ryff’s scales of Psychological Well-being (Ryff, 1989). These scales measure six dimensions of well-being: self-acceptance, positive relations with others, autonomy, environmental mastery, purpose in life, and personal growth. The 18-item version developed by Ryff and Keyes (1995) was used. Well-being subscales were clustered into two dimensions of well-being, following Keyes et al. (2002) work: psychological well-being (autonomy, purpose in life, personal growth and positive relations) and subjective well-being (environmental mastery and self-acceptance). Internal consistency estimates were 0.75 and 0.74, respectively.
(b) From the 42-item Spanish version (Sandín and Chorot, 2003) of the Coping Strategies Questionnaire (CSQ; Rosenstiel and Keefe, 1983), a scale measuring seven coping strategies (negative auto-focused, positive reappraisal, avoidance, religious, problem-solving coping, overt emotional expression, and social support seeking), the three items with higher loadings of each dimension were chosen. These dimensions are thought to be part of two higher order dimensions: problem-focused coping, including problem-solving, positive reappraisal and social support seeking; and emotion-focused coping strategies, including negative auto-focused coping, overt emotional expression, avoidance, and religious coping (Sandín and Chorot, 2003). Internal consistency estimations were 0.81 and 0.77, respectively.
(c) Short Form-8 Health Survey Questionnaire (Ware et al., 2001), an 8-item long scale designed to monitor population health that provides a general measure of both mental and physical health. Internal consistency estimate was 0.91.

### Statistical Analyses

A set of competitive full SEM were tested to study the role of problem- and emotion-focused coping strategies on well-being. Several fit indexes were obtained: chi square statistic (χ^2^), Comparative Fit Index (CFI), Root Mean Squared Error of Approximation (RMSEA), Standardized Root Mean Squared Residual (SRMR), Akaike’s Information Criterion (AIC), and Goodness of Fit Index (GFI). All models were estimated with Maximum Likelihood Robust (MLR). A model is considered to display good fit when CFI is above 0.90 and RMSEA is below 0.08 (Hu and Bentler, 1999). Analyses were performed using EQS 6.1.

## Results

An initial set of six structural models were tested. Models 1.1. to 1.3. modeled a single factor of well-being; models 2.1 to 2.3 modeled two factors of well-being:

• Model 1.1. Two factors of coping strategies were proposed, based on the distinction between emotion- and problem-focused coping strategies defined by citeBR20. These factors explained a general factor of well-being, as theoretically postulated by Ryff (1995).
• Model 1.2. Problem- and emotion-focused coping were maintained, but problem-solving coping and avoidance coping were explained by both factors of coping strategies, which accounted for a general factor of well-being. This modification was analogous to the cross-loadings of coping strategies in Mayordomo-Rodríguez et al. (2015a).
• Model 1.3. The religious coping dimension was removed, and problem- and emotion-focused coping explained a general factor of well-being. In the work by Mayordomo-Rodríguez et al. (2015a) good fit was achieved when removing the religious coping dimension.
• Model 2.1. Two factors of coping strategies were proposed, based on the distinction between emotion and problem-focused coping by Lazarus and Folkman (1984), which explained two factors (psychological and subjective) of well-being as exposed in Keyes et al. (2002).
• Model 2.2. The dimensions of problem-solving coping and avoidance coping were explained by both problem- and emotion-focused coping, which accounted for psychological well-being and subjective well-being. This was similar to the cross-loadings of coping strategies in Mayordomo-Rodríguez et al. (2015a).
• Model 2.3. The religious coping dimension was removed, leaving the remaining dimensions defined by problem- and emotion-focused coping, which at the same time explained the psychological and subjective well-being factors. Removing religious coping yielded good fit of a model in Mayordomo-Rodríguez et al. (2015a).

Model fit indexes for the first set of models are shown in Table 2. Both Model 1.1 and Model 1.2 showed an acceptable RMSEA index, but CFI indices indicated the models had not an acceptable fit to the data. Model 1.3, in turn, complied with criteria for both RMSEA and CFI indices.

**TABLE 2:** Fit indices for all tested models.

	χ^2^	*df*	*p*	CFI	GFI	RMSEA	90% CI	SRMR	AIC
Model 1.1	301.721	62	<0.001	0.824	0.932	0.071	[0.063,0.079]	0.075	177.721
Model 1.2	230.054	60	<0.001	0.875	0.947	0.061	[0.053,0.069]	0.061	110.055
Model 1.3	178.463	49	<0.001	0.900	0.954	0.058	[0.049,0.067]	0.052	80.463
Model 2.1	290.158	59	<0.001	0.830	0.935	0.072	[0.063,0.080]	0.074	172.158
Model 2.2	218.711	57	<0.001	0.881	0.950	0.061	[0.052,0.070]	0.060	104.712
Model 2.3	165.910	46	<0.001	0.906	0.957	0.058	[0.048,0.067]	0.050	73.911

**FIGURE 1:**
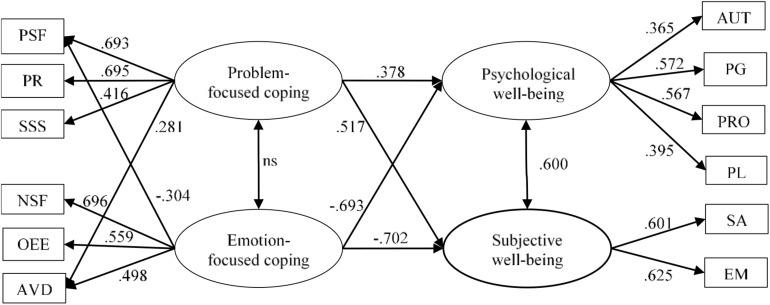
Best-fitting model from the second set of models, and from all tested models. All shown values are significant at 0.05 level. ns = not significant. See Table 1 for abbreviations.

Results of the second set of tested models are shown in Table 2. Again, Model 2.1 and Model 2.2 failed to comply with criteria for the CFI index, although the RMSEA values were acceptable. Model 2.3 was the best fitting model, even when compared with 1.3: lower AIC and an increase of the CFI. Factor loadings of the best-fitting model (Model 2.3) are shown in Figure 1.

**FIGURE 2:**
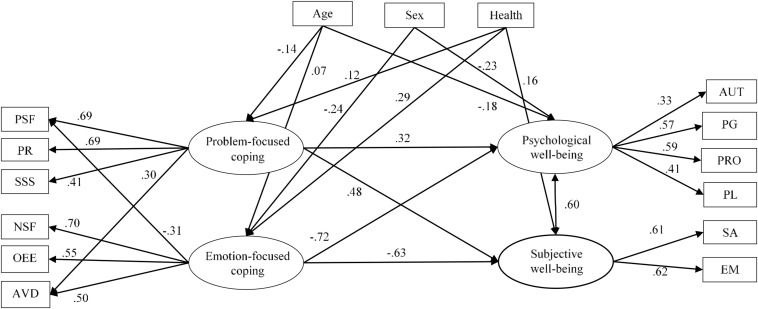
Final structural model with control variables. All values are significant at 0.05 level. See Table 1 for abbreviations.

Once a best-fitting model was established, a number of variables were entered to control for their potential effects on the model: age, sex, being a widow or widower and perceived health status. This new model fitted the data well: χ^2^(79) = 263.25, *p* < 0.001; RMSEA = 0.055 [0.048,0.063]; CFI = 0.890; SRMR = 0.049; GFI = 0.951. Nevertheless, some of the effects of the control variables were statistically non-significant and these relationships were removed (including all the effects of widowhood) A new more parsimonious model was tested, which fitted the data better: χ^2^(77) = 250.8, *p* < 0.001; RMSEA = 0.055 [0.048,0.063]; CFI = 0.902; SRMR = 0.050; GFI = 0.960. Parameter estimates are presented in Figure 2. Problem-focused coping had significant effects on both well-being dimensions, while emotion-focused had negative effects. Regarding the control variables, age had a negative effect on problem-focused coping and a positive effect on emotion-focused coping, men used less emotion-focused coping, and health had a positive impact on the use of both dimensions of coping. Control variables were also related to well-being: age negatively predicted psychological well-being, men had less psychological well-being, and health positively predicted subjective well-being.

## Discussion

The aim of this study was to better understand the role of coping strategies in old adults’ well-being, through a set of competitive SEMs partially based on Mayordomo-Rodríguez et al. (2015a). In this model, two factors of coping strategies, problem- and emotion-focused coping, explained a general factor of well-being in a sample of young adults.

Regarding the retained, best-fitting, model, religion strategy was removed because it did not load onto any of the two coping factors and two cross-loadings were included. Thus, the structure of the coping strategies here is pretty similar to the one posed by Mayordomo-Rodríguez et al. (2015a), who also dismissed the religious coping dimension and posed two cross-loaded dimensions. Our two cross loadings seem theoretically reasonable. On the one hand, problem-solving strategy loaded negatively in the emotion coping factor, that is, those old people using more emotion-focused strategies displace the use of problem solving coping. On the other hand, avoidance coping may be thought as a reasonable problem-focused strategy in the old age in light of SOC theory (Baltes and Baltes, 1990), as selection is considered a relevant and adequate coping strategy in this model. The relationship between the two factors of coping strategies, problem- and emotion-focused, resulted non-significant. This is in line with Mayordomo-Rodríguez et al. (2015a) results, although somehow contradicts previous literature (Lazarus, 2006; Tomás et al., 2012). When studied in old adults, problem-focused and emotion-focused coping strategies have showed a positive relation. Tomás et al. (2012), for example, found a positive and moderate relation between the two types of coping, supporting Lazarus (2006) idea that they were complementary strategies. Later evidence gathered by Mayordomo-Rodríguez et al. (2015a), however, is more similar to the original Folkman’s theory, signaling toward two distinct and independent dimensions, which is corroborated by our results.

When it comes to the well-being measurement part, models showed slightly better fit every time well-being was conceptualized as two different dimensions: psychological and subjective well-being. This is in line with the conceptualization of Ryff’s dimensions by Keyes et al. (2002). Evidence of current research supports this distinction, both by means of measurement adequacy of the model, and by predictive effects of coping strategies, which differed between the two dimensions of well-being.

Regarding the predictive part of the model, our results agree with our general hypotheses: problem-focused coping had a positive predictive effect on well-being while emotion-focused showed a negatively one. When compared to Mayordomo-Rodríguez et al. (2015a) work, there are some similarities but also differences. Among the differences we highlight the design of the study. While Mayordomo-Rodríguez et al. (2015a) explored the effect of the different strategies on a general dimension of well-being, current research considered two approaches to well-being, the unidimensional and the multidimensional one, which allowed to disentangle different effects. Indeed, both problem-focused and emotion-focused had stronger relations with subjective well-being, compared to psychological well-being. Nevertheless, these hypotheses were tentative, and based in previous results by Mayordomo-Rodríguez et al. (2015a) and meta-analytic evidence from Penley et al. (2002), as previously stated. As PCP model predicted that emotion-focused strategy may also add to resources, our results partially contradict this theoretical model. Therefore, the different effects of these strategies as well as the mechanism behind the effect of copying strategies on well-being must be better understood.

Four main ideas can be drawn from these results. First, both problem- and emotion-focused coping strategies were statistically significant predictors of both subjective and psychological well-being. Although this relationship between coping and well-being has been found in different samples such as women with breast cancer (Kim et al., 2010); nursing students (Por et al., 2011); or adolescents (Griffith et al., 2000; Cicognani, 2011), it has not been exhaustively studied in old age (Tomás et al., 2012). This study, then, posits the relevance of coping strategies for old people’s achievement of optimal levels of well-being. The second and third contributions of this paper are related to the differential role of coping dimensions in the prediction of well-being. Emotion-focused strategies had more predictive power on well-being. And whereas problem-focused strategies positively predicted both subjective and psychological well-being, emotion-focused strategies negatively predicted them. This is also in line with previous results in adolescent literature, which linked active coping styles to greater well-being, whereas avoidance had been linked to poor adaptation and higher levels of depressive symptoms (Griffith et al., 2000; Cicognani, 2011). This may be considered a main contribution of this research as it partially challenges the PCP model. This model considers all coping strategies as a personal resource that ought to contribute to positive outcomes in aging. By showing that the effects of emotion-focused strategies were negatively associated with well-being, our results make evident the necessity to better understand when and how old people use these emotion-focused strategies and to disentangle the mechanism that leads to negative outcomes. For instance, Cicognani (2011) found that well-being was increased by lower use of withdrawal coping strategies, which could be assimilated to the dimensions of overt emotional expression and avoidance of this study, that is, to the emotion-focused coping strategies. In older adults’ context, similar results were offered by Tomás et al. (2012). These results are no good news because literature has shown that these emotion-focused strategies rise in the old age. Finally, and regarding the predictive power of the model, evidence yielded to a higher proportion of explained variance of psychological well-being compared to subjective well-being. It is a reasonable result, as subjective well-being is related to the existential challenges, whereas psychological well-being refers to affect or quality of life (Keyes et al., 2002). All in all, interventions carried out to improve older adults’ well-being should put their focus on minimizing the use of emotion-coping strategies, while promoting problem-focused strategies for old people when possible.

Additionally, the control variables gave additional information. Health and age had expected effects, but the results of sex were somehow inconsistent with the literature. It is well-established that women tend to use coping strategies that are aimed at changing their emotional responses to a stressful situation, whereas men use more problem-focused or instrumental methods of handling stressful experiences (Endler and Parker, 1990; Ptacek et al., 1994; Kelly et al., 2008). This use of emotion-coping strategies could be one reason why women tend to report more psychological distress, symptoms of depression and anxiety than men, and this could lead to worse well-being (Kuehner, 2003; Mazure and Maciejewski, 2003; Matud, 2004). Indeed, the literature shows that women who use more emotion-focused coping styles report more depressive and anxiety-related symptoms than women who show lower frequency of use of emotion-focused coping strategies (Cohen, 2002; Bennett et al., 2005). Contrarily to this, women in our study reported more psychological well-being.

These results have to be cautiously interpreted, as this research presents some shortcomings, such as the specificity of the population assessed (attendants to a university long-life learning program), bias due to social desirability, or its cross-sectional design. In this regard, the generalizability of our results to general, older people population could be hinted. As regards social desirability, and taking into account that well-being is a social desirable outcome, specifically in countries such as Spain, controlling for this variable could have helped eliminate possible biases. When it comes to the cross-sectional design, and although this study was based on previous literature (Tomás et al., 2012; Mayordomo-Rodríguez et al., 2015a) which has specified the prediction of coping strategies on well-being, it could also be possible that it was the latter that affected the former. Therefore, future studies in general population, controlling for social desirability and with a longitudinal design, would be welcomed.

## Data Availability Statement

The raw data supporting the conclusions of this manuscript will be made available by the authors, without undue reservation, to any qualified researcher.

## Ethics Statement

The studies involving human participants were reviewed and approved by University of Valencia Ethics Committee. The patients/participants provided their written informed consent to participate in this study.

## Author Contributions

LG and IF worked in the introduction, results, and discussion. JT and AO worked in the research design, results, and discussion.

## Conflict of Interest

The authors declare that the research was conducted in the absence of any commercial or financial relationships that could be construed as a potential conflict of interest.
