# Corrigendum: Predicting Well-Being Among the Elderly: The Role of Coping Strategies

**DOI:** 10.3389/fpsyg.2020.02125

**Published:** 2020-09-04

**Authors:** Laura Galiana, José M. Tomás, Irene Fernández, Amparo Oliver

**Affiliations:** Department of Methodology of the Behavioural Sciences, University of Valencia, Valencia, Spain

**Keywords:** problem-focused coping strategies, emotion-focused coping strategies, psychological well-being, subjective well-being, elderly

There is an error in the Funding statement. The correct Funding section should read as follows:

“This work was partially supported by grant UV-INV-AE18-777619 from the University of Valencia and funded by Project RTI2018-093321-B-100 (FEDER/Ministerio de Ciencia e Innovación – Agencia Estatal de Investigación, Spain).”

The authors apologize for this error and state that this does not change the scientific conclusions of the article in any way. The original article has been updated.

